# Comment on: “Treatment of idiopathic granulomatous mastitis and factors related with disease recurrence”

**DOI:** 10.3906/sag-2005-334

**Published:** 2020-12-17

**Authors:** Sami AKBULUT, Tevfik Tolga ŞAHİN

**Affiliations:** 1 Department of Surgery and Liver Transplant Institute, İnönü University Faculty of Medicine, Malatya Turkey

**To the Editor** ,

We read the recent article “Treatment of idiopathic granulomatous mastitis and factors related with disease recurrence” published by Tekgoz and colleagues with great interest [1]. The authors stated that they aimed to investigate the effect of immunosuppressive treatment on idiopathic granulomatous mastitis (IGM) and risk factors related to IGM recurrence.

We would like to emphasize a few important points regarding the medical management algorithm of the present study.

The authors state that they have applied a cross-sectional retrospective research model in the present study. This statement is wrong in terms of epidemiological aspect because the studies that go from the outcome to the exposure are retrospective case-control studies. The cross-sectional studies evaluate the exposure and outcome at the same time and are usually performed to determine the prevalence of certain conditions or diseases. Other names of the study method are epidemiological surveillance, transvers study, screening, fact-findings survey, ad hoc survey, point-in-time study, and one-shot survey.

The authors did not give any information regarding their management protocol to patients with IGM. In Turkey, the duration from admission to the histopathological diagnosis of IGM is around 3–4 weeks. In patients with abscess and cellulitis, it is not rational to wait for the biopsy result without performing drainage and/or antibiotics. The current literature suggests that drainage and antibiotics should be performed to patients admitted with abscess and cellulitis. Also, patients with inflammatory mass should receive antiinflammatory treatment after the biopsy until the definitive results are obtained. We would like to share our diagnostic and therapeutic algorithm that we have deduced from our own experiences and extensive review of the literature (Figure). We believe it will contribute to the present study and this extensive algorithm is first in the medical literature.

**Figure:**
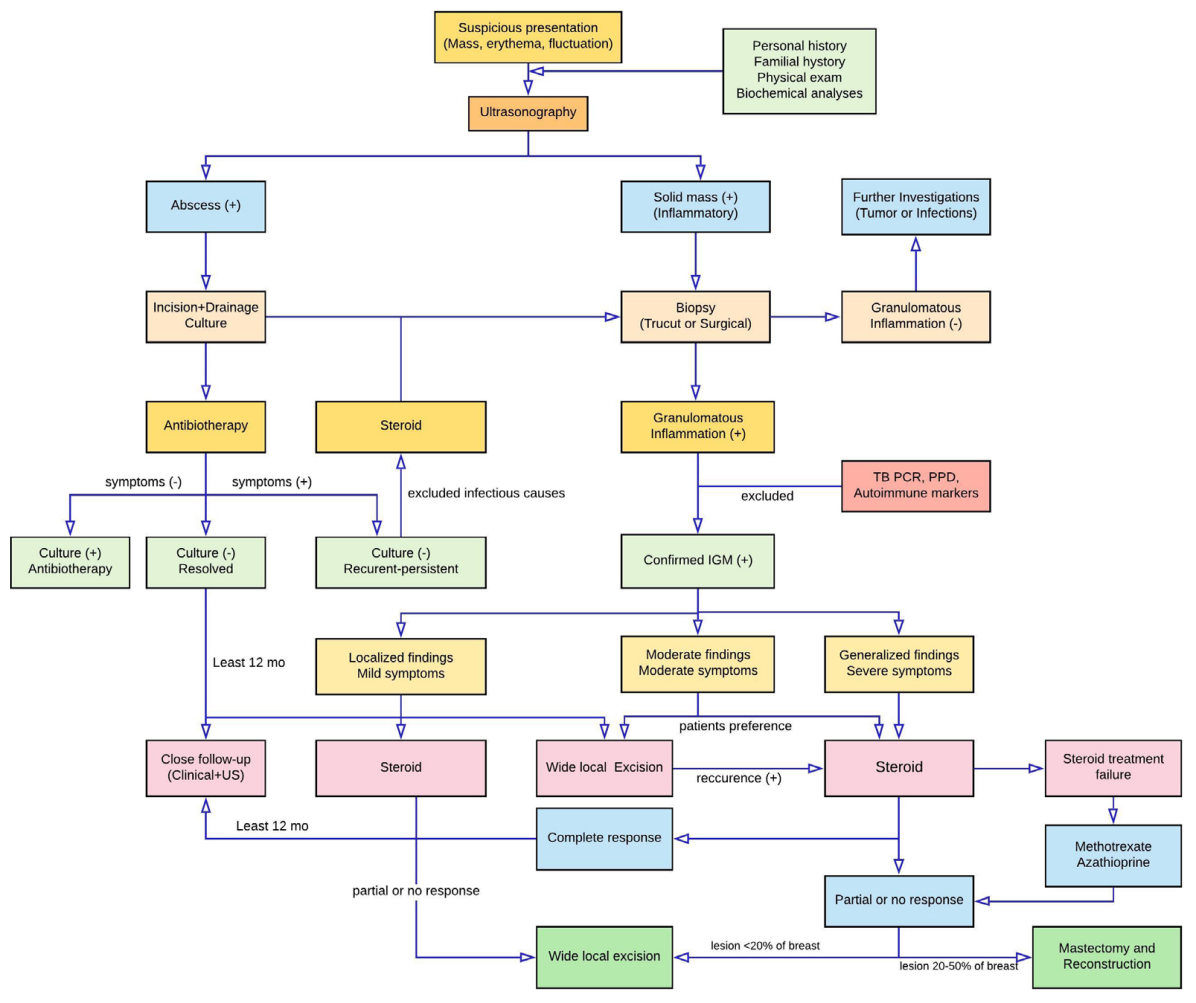
Diagnostic and therapeutic management algorithm for patients with IGM.

The authors state that they have given methotrexate (MTX; 10–15mg/week) treatment for a median of 9.1 months together with concomitant steroid therapy (40 mg/day) for a median of 9.6 months to 96.2% of the patients. As we have contributed significantly and among the first surgeons who have used MTX in the treatment of IGM, we recommend that the first-line therapy of IGM should always begin with steroid therapy [2,3]. However, we recommend the administration of MTX occasionally azathioprine in patients who develop complications (such as glucose intolerance, hypertension, central obesity, and osteoporosis) due to steroid therapy, in patients with poor response to steroid therapy, or in patients who develop remission due to steroid therapy but develop recurrence after discontinuation of steroid therapy. However, MTX can be initiated as a first-line therapy in patients with contraindications that use steroid therapy, but MTX therapy should be initiated at a dose of 5 mg/week and the side effect of the drug should be checked by routine blood tests. Briefly, only a few researchers suggest the use of MTX or MTX plus steroid as the first-line therapy; however, there is a consensus on the use of steroid therapy before initiating MTX [2,4].

Long-term therapy of MTX and steroids for completely benign disease should be evaluated carefully due to its detrimental effects in patients with an age range of 20–40 years. Besides nearly 29–50% recurrence rates following steroids or MTX treatment result in questioning the necessity of steroids or MTX therapy in patients [4,5]. Therefore, one of the radical surgical treatment options (partial or total mastectomy-plus breast reconstruction) should be offered to reproductive aged-patients who have recurred IGM disease despite more than 2 cycles of steroid and MTX treatment. Since it is necessary to inform the patients correctly about the side effects of long-term immunosuppressive treatment protocols.

The authors have made a misfortunate statement that none of the imaging studies have any superiority over biopsy. Pathologic analysis is a definitive diagnostic tool and expecting any other adjunctive diagnostic study to be superior to a gold standard test is not logical. However, studies can determine the sensitivity, specificity, and predictive value of a given test per the gold standard reference test which is histopathologic analysis.
